# A Thoracic Endometriosis-Related Catamenial Hemopneumothorax in a Woman With Premature Ovarian Failure

**DOI:** 10.7759/cureus.17110

**Published:** 2021-08-11

**Authors:** Deepak P Kalbi, Ali F Al Sbihi, Nouraldeen Manasrah, Ahmed J Chaudhary, Sana Iqbal

**Affiliations:** 1 Internal Medicine, Detroit Medical Center Sinai-Grace Hospital, Detroit, USA; 2 Nuclear Medicine, Montefiore Medical Center, New York, USA

**Keywords:** thoracic endometriosis syndrome, premature ovarian failure, catamenial hemopneumothorax, endometriosis, exertional dyspnea

## Abstract

Endometriosis is the presence of endometrial glands and stroma outside the uterine cavity. It is usually confined to the pelvis, particularly the ovaries, cul-de-sac, broad ligaments, and uterosacral ligaments, but it can also expand outside the pelvis. The thorax is among the common extrapelvic locations. Thoracic endometriosis syndrome (TES) is a rare disorder characterized by the presence of functioning endometrial tissue in the pleura, lung parenchyma, and airways. This report presents a case of a young female patient with advanced endometriosis and premature ovarian failure who was admitted with dyspnea that turned to be due to a rare endometriosis-related complication.

## Introduction

Thoracic endometriosis-related hemopneumothorax (TERP) or thoracic endometriosis syndrome (TES) usually occurs in childbearing age women and affects the right thorax. Cases that occur after menopause and left-sided cases are rarer [1]. Catamenial pneumothorax is a specific condition that occurs in conjunction with menstrual cycles or during ovulation. Clinicians should be familiar with rare endometriosis complications in order to have early diagnosis and treatment.

## Case presentation

A 27-year-old African American female patient with a history of stage IV endometriosis presented to the emergency department with exertional dyspnea of three-month duration. She also had an unintentional weight loss of approximately 25 pounds. The patient also endorsed night sweats, paroxysmal nocturnal dyspnea, and orthopnea, but she denied chest pain or lower extremity swelling. Of notice, the patient had an endometrial cyst removal performed five years ago and an endometriosis-related paracentesis performed three years ago. The last follow-up with obstetrics/gynecology was 13 months ago.

On presentation, the patient had a blood pressure of 120/78 mmHg, a temperature of 36.6°C, a heart rate range of 75-90 beats per minute, and a respiratory rate of 16 breaths per minute with an oxygen saturation of 97% on room air. Chest auscultation revealed absent breath sounds on the right side of the chest.

**Figure 1:**
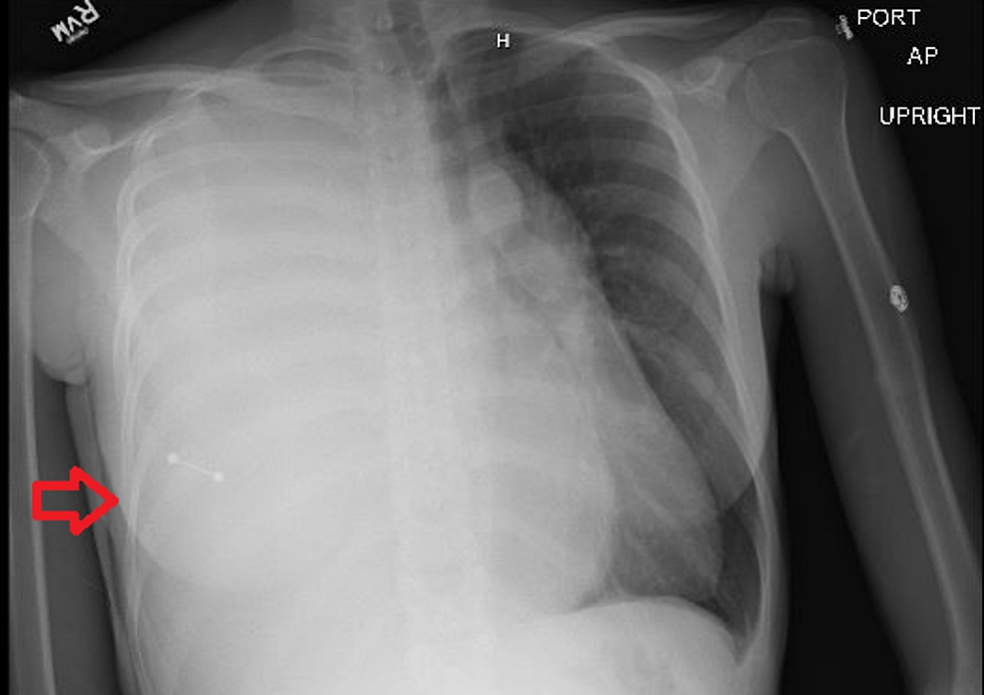
CXR on admission day showed complete opacification of the right side (red arrow) with a left-sided tracheal deviation AP: anterior-posterior, CXR: chest X-ray, H: hilum and mediastinum, Rvm: Relevance Vector Machine.

**Figure 2:**
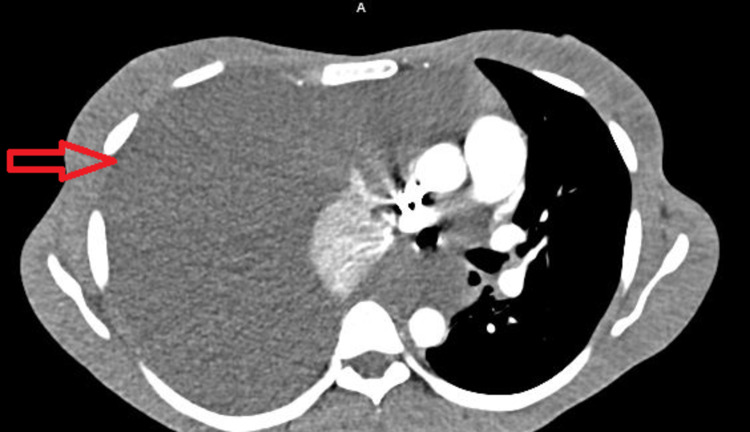
CT thorax of pulmonary arteries showed massive right pleural effusion with complete atelectasis of the right lung (red arrow) in addition to a left-sided cardiomediastinal shift A: anterior.

**Figure 3:**
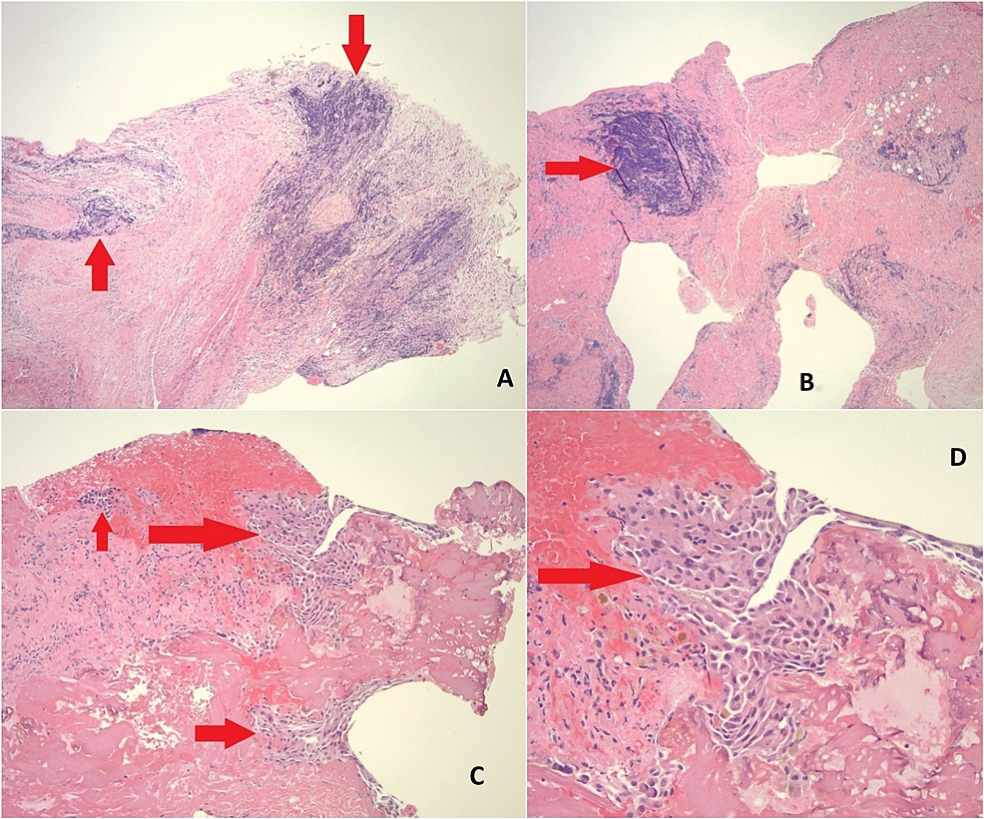
Pathology slides A: Microscopy of diaphragm biopsy (4× magnification) showing chronic inflammation with lymphoid aggregates (red arrows). B: Microscopy of pleural biopsy (4× magnification) showing chronic inflammation with lymphoid aggregates (red arrow). C: Microscopy (10× magnification) showing mesothelial hyperplasia (red arrows). D: Microscopy (20× magnification) showing mesothelial hyperplasia (red arrow).

Laboratory studies were significant for a hemoglobin of 11.2 g/dl (12-16 g/dl). Other complete blood count parameters and electrolytes were within normal limits. Chest X-ray (CXR) showed complete opacification of the right chest with a left tracheal deviation (Figure 1). Computed tomography of pulmonary arteries (CT-PA) was negative for pulmonary emboli, but it showed a massive right pleural effusion with complete atelectasis of the right lung in addition to a left-sided cardiomediastinal shift (Figure 2). Thoracocentesis with the removal of 2 to 3 l of serosanguinous fluid was performed, fluids turned to be exudative with few glandular and endometrial cells on cytohistological examination. Because the patient’s condition was not improving, a chest tube was placed. Continuous chest tube drainage with serosanguinous fluid was noted for eight days. Because the patient’s condition did not significantly improve, a right video-assisted thoracoscopic surgery (VATS), a right diaphragm and pleural biopsy, and pleurodesis were performed, which revealed dense fibroconnective tissue with prominent chronic inflammation, including lymphoid aggregates and focal mesothelial hyperplasia (Figure 3). No malignancy was reported in the histopathologic examination.

**Figure 4:**
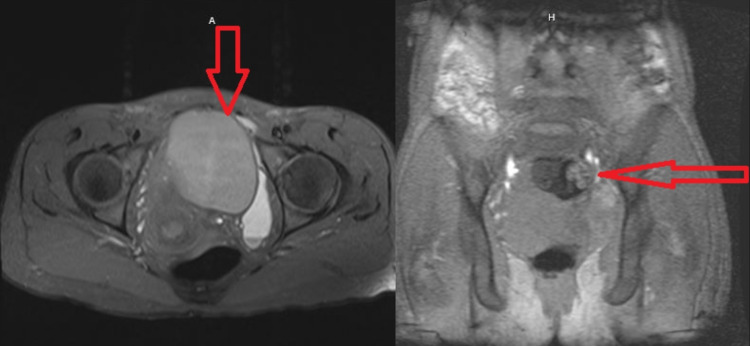
MRI chest/abdomen/pelvis MRI findings suggestive of an endometrioma measuring 7.2 × 7.1 × 4.2 cm in the mid-upper pelvis abutting and pushing the uterus to the right (red arrow, left). Moderate amount of hemorrhagic fluid in the pelvis and bilateral paracolic gutters with loculated left pelvic fluid measuring 9.3 × 3.3 cm (red arrow, right). A: anterior, H: hilum and mediastinum.

Ultrasonography of the abdomen and pelvis in addition to magnetic resonance imaging (MRI) of the thorax, abdomen, and pelvis was done; these investigations showed endometrioma of about 7.2 × 7.1 × 4.2 cm in the mid-upper pelvis with a moderate amount of hemorrhagic fluid in the pelvis and bilateral paracolic gutters (Figure 4), and the latter finding was similar to a prior finding on a CT scan of the abdomen that was done three years ago. Because these fluids did not cause any symptoms to the patient, the obstetrics/gynecology team’s decision was not to drain, believing that the endometriosis treatment itself should improve this condition.

**Figure 5:**
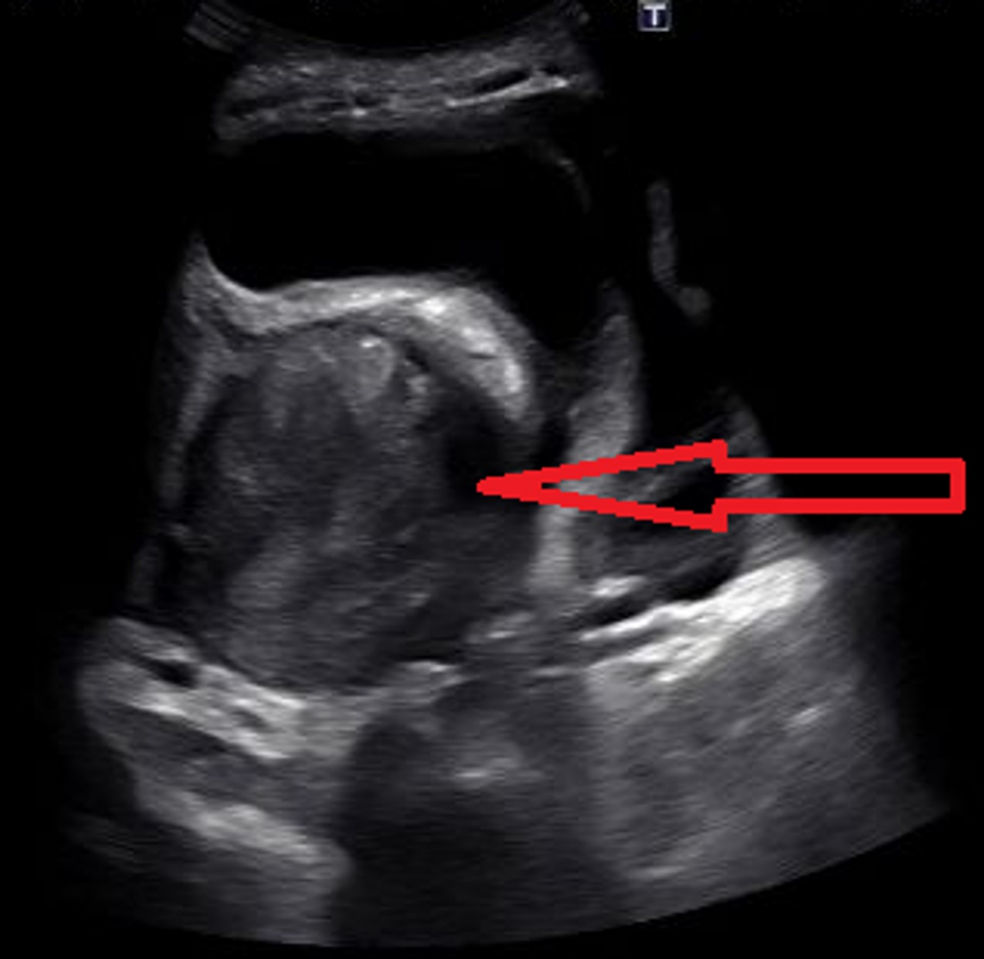
Transvaginal ultrasound showing a large septate cystic lesion in the left adnexa (red arrow), which has an appearance consistent with endometrioma T: transvaginal.

Transvaginal ultrasonography and complete pelvic duplex ultrasonography of the lower abdomen showed a large septate cystic lesion in the left adnexa, which has been present for three years. The lesion was consistent with endometrioma (Figure 5). The patient was discharged on Leuprolide Depot 11.25 mg injections every three months and norethindrone hormonal therapy of 5 mg tablets daily to prevent menopausal symptoms, with a close outpatient follow-up.

## Discussion

Endometriosis is defined as the presence of endometrial glands and stroma outside the uterine cavity and is usually confined to the pelvis, particularly the ovaries, cul-de-sac, broad ligaments, and uterosacral ligaments. However, endometrial tissue can be found outside the pelvis in the abdomen, thorax, brain, and skin [1]. TES is a rare disorder characterized by the presence of functioning endometrial tissue in the pleura, lung parenchyma, and airways. Thoracic involvement is the most frequent extrapelvic location of endometriosis.

One retrospective study on TES included 110 patients and showed that the mean age at presentation was 35 ± 0.6 years, ranging from 15 to 54 years [2]. Surprisingly, the peak incidence for pelvic endometriosis in the patients of this study occurred between 24 and 29 years, whereas the peak incidence for TES occurred approximately five years later.

Three theories have been presented to explain the presence of intrathoracic endometrial implants: coelomic metaplasia, lymphatic or hematogenous embolization from the uterus or pelvis, and retrograde menstruation with subsequent transperitoneal-transdiaphragmatic migration of endometrial tissue. None of these theories can clarify all the clinical manifestations of TES, and the disease likely has a multifactorial etiology [2,3].

Catamenial pneumothorax is a spontaneous recurrent pneumothorax during menstruation. It is the most common presentation of TES [4]; it is responsible for only 2.5% to 5% of cases of women with spontaneous pneumothorax, and the right hemithorax is involved in most cases [3].

Catamenial pneumothorax is usually characterized by shortness of breath or chest or shoulder pain occurring 24 hours before to 72 hours after menses. It can be a challenging diagnosis. Even in cases where the gold standard test (VATS) is performed, uncertainty continues to be a major issue. Symptoms consistent with catamenial pneumothorax, despite a negative tissue diagnosis, should prompt hormonal treatment, and the clinician should always have a low threshold for evaluation for recurrent pneumothorax in these patients [5,6]. VATS usually provides good diagnostic and therapeutic results, but a good percentage of patients experience recurrence. In one study, it was noted that 25% of patients had a recurrence, despite adequate treatment with VATS [7].

Catamenial pneumothorax with mild symptoms is usually managed with simple rest and thoracocentesis or chest tube for symptomatic relief [8]. Surgery is usually performed when conservative treatment fails or in women with multiple recurrences. Most surgical treatments involve thoracoscopy and pleurodesis to reduce recurrences. Blebectomy or apical wedge pleurectomy has also been done [9].

A systemic review conducted in 2020 concluded that postoperative treatment with a recently developed gonadotropin-releasing hormone (GnRH) agonists can be given intramuscularly (IM) or orally, or oral contraceptives similar to that given for pelvic endometriosis can also be administered for symptom relief, reduce recurrences, and suppress concomitant pelvic endometriosis [6]. Ovarian suppression with GnRH agonists and add-back treatment will mitigate the adverse effects of menopausal symptoms [10].

## Conclusions

Thoracic endometriosis is an underrecognized and not fully understood condition. It is often diagnosed late after the presentation. TES is a rare and complex condition. It requires a high index of suspicion in any woman of reproductive age or those who are receiving hormone replacement therapy. Common symptoms are cyclical chest pain, dyspnea, and/or hemoptysis. Our case report increases the awareness among healthcare practitioners to have a low threshold of suspicion for a rare complication of endometriosis to allow for early diagnosis and avoid further complications that could be fatal if not addressed earlier.
